# Apparent mineralocorticoid excess caused by novel compound heterozygous mutations in *HSD11B2* and characterized by early-onset hypertension and hypokalemia

**DOI:** 10.1007/s12020-020-02460-9

**Published:** 2020-08-20

**Authors:** Peng Fan, Yi-Ting Lu, Kun-Qi Yang, Di Zhang, Xue-Ying Liu, Tao Tian, Fang Luo, Lin-Ping Wang, Wen-Jun Ma, Ya-Xin Liu, Hui-Min Zhang, Lei Song, Jun Cai, Ying Lou, Xian-Liang Zhou

**Affiliations:** 1 Department of Cardiology, Fuwai Hospital, National Center for Cardiovascular Diseases, Chinese Academy of Medical Sciences and Peking Union Medical College, Beijing, China; 2 Emergency and Critical Care Center, Fuwai Hospital, National Center for Cardiovascular Diseases, Chinese Academy of Medical Sciences and Peking Union Medical College, Beijing, China

**Keywords:** Apparent mineralocorticoid excess, Compound *HSD11B2* mutations, Early-onset hypertension, Hypokalemia

## Abstract

**Purpose:**

Apparent mineralocorticoid excess (AME) is an ultrarare autosomal recessive disorder resulting from deficiency of 11β-hydroxysteroid dehydrogenase type 2 (11βHSD2) caused by mutations in *HSD11B2*. The purpose of this study was to identify novel compound heterozygous *HSD11B2* mutations in a Chinese pedigree with AME and conduct a systematic review evaluating the AME clinical features associated with *HSD11B2* mutations.

**Methods:**

Next-generation sequencing was performed in the proband, and Sanger sequencing was used to identify candidate variants in family members, 100 hypertensives, and 100 healthy controls. A predicted structure of 11βHSD2 was constructed by in silico modeling. A systematic review was used to identify cases of *HSD11B2*-related AME. Data for genotyping and clinical characterizations and complications were extracted.

**Results:**

Next-generation sequencing showed novel compound heterozygous mutations (c.343_348del and c.1099_1101del) in the proband with early-onset hypertension and hypokalemia. Sanger sequencing verified the monoallelic form of the same mutations in five other relatives but not in 100 hypertensives or 100 healthy subjects. In silico structural modeling showed that compound mutations may simultaneously perturb the substrate and coenzyme binding pocket. A systematic review of 101 AME patients with 54 *HSD11B2* mutations revealed early-onset hypertension, hypokalemia and homozygous mutations as common features. The homozygous *HSD11B2* mutations correlated with low birth weight (*r* = 0.285, *P* = 0.02).

**Conclusions:**

We report novel compound heterozygous *HSD11B2* mutations in a Chinese teenager with early-onset hypertension, and enriched genotypic and phenotypic spectrums in AME. Genetic testing helps early diagnosis and treatment for AME patients, which may avoid target organ damage.

## Introduction

Apparent mineralocorticoid excess (AME, OMIM #218030) is an ultrarare autosomal recessive form of monogenic hypertension. Although the biochemical and hormonal features of AME were first described in 1977 by New et al. [1], the first causative mutation in *HSD11B2* was not discovered until 1995 in a consanguineous Iranian family with AME [2]. Since then, only ~100 AME cases have been described clinically and genetically worldwide, and the prevalence of AME remains uncertain. However, AME is most commonly found in consanguineous families or certain ethnic groups [3–5].

AME is characterized by juvenile hypertension, hypokalemia, hypernatremia, low plasma renin activity and aldosterone concentration, metabolic alkalosis, and responsiveness to spironolactone [6]. It has a spectrum of phenotypes ranging from life-threatening hypertension in infancy to a milder form of the disease in adults [7]. Because of the broad diversity and overlapping clinical features, precise diagnosis of AME is highly reliant on genetic evidence [8].

AME is caused by a mutation in *HSD11B2*, which has been mapped to chromosome 16q22 and consists of five exons. It encodes 11β-hydroxysteroid dehydrogenase type 2 (11βHSD2), which is a microsomal enzyme mainly expressed in mineralocorticoid target tissues, such as distal nephron [9]. 11βHSD2 plays an important role in the peripheral inactivation of cortisol to cortisone, thus protecting the mineralocorticoid receptor (MR) from inappropriate activation by cortisol [10]. *HSD11B2* mutations lead to a deficiency in the 11βHSD2 enzyme [11], resulting in excessive cortisol stimulating the MR and causing intense water and sodium retention, hypokalemia, and hypertension [12].

This study aimed to identify novel compound heterozygous mutations in *HSD11B2* in a Chinese family with AME. We constructed a computational model of 11βHSD2 to evaluate genotype-structure-phenotype correlations. We also conducted a systematic review to summarize the clinical features of AME patients associated with loss-of-function *HSD11B2* mutations.

## Materials and methods

### Subjects

**Fig. 1:**
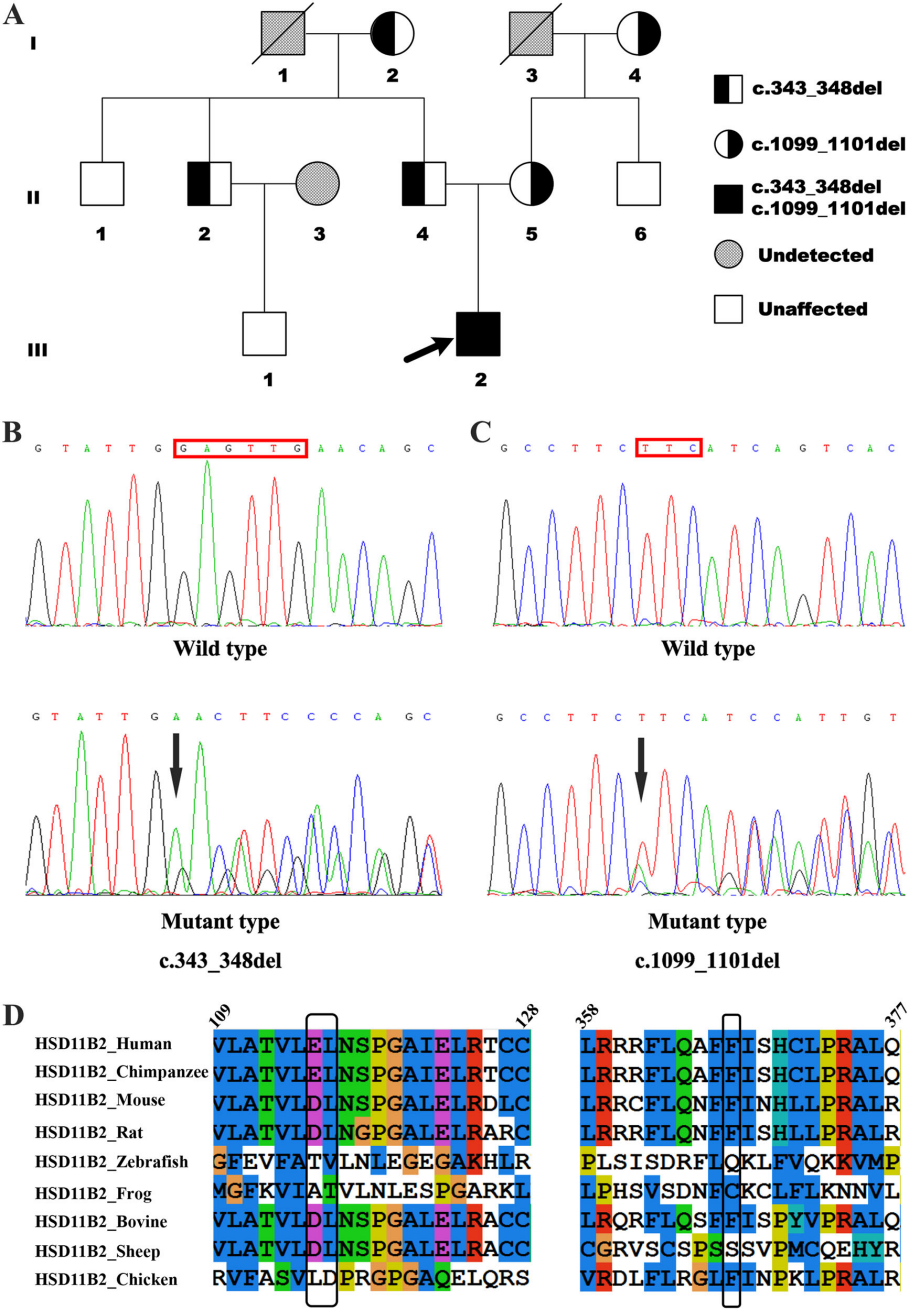
Novel compound heterozygous mutations in *HSD11B2* cause apparent mineralocorticoid excess syndrome in a Chinese family. **a** Pedigree chart of this family. **b**, **c** Sanger sequencing of healthy individuals and subjects carrying c.343_348del and c.1099_1101del mutations, respectively. **d** Sequence analysis of *HSD11B2* showing conservation of the identified compound mutations

The 17-year-old male proband (subject III-2), presented with a 2-year history of hypertension (fluctuating from 150/90 to 170/100 mmHg) and hypokalemia (fluctuating from 2.87 to 3.15 mmol/L). Because of no symptoms of discomfort, he had not undergone further examination or treatment. Two months previously, he first experienced headache, dizziness, and fatigue, together with an elevated blood pressure (BP; 180/110 mmHg) and decreased plasma potassium (2.23 mmol/L). He was born to a non-consanguineous family after a full-term (37-week) pregnancy with a normal birth weight (3.2 kg), and physical and psychomotor development was normal. He had no history of licorice ingestion. He was admitted to Fuwai Hospital to identify the etiology of early-onset hypertension. Eight other family members (Fig. 1a), 100 hypertensives, and 100 healthy controls were also enrolled in this study.

This study was approved by the Ethics Committee of Fuwai Hospital and carried out in accordance with the principles set out in the Declaration of Helsinki. Each participant provided written informed consent.

### Biochemical evaluation

The plasma renin concentration and plasma aldosterone concentration (PAC) were measured by chemiluminescence immunoassay using the LIAISON® direct renin kit (DiaSorin S.p.A, Vercelli, Italy) and the LIAISON® aldosterone kit (DiaSorin Inc., Stillwater, MN, USA). Other biochemical examinations were performed using standard protocols.

### Genetic sequencing

Genomic DNA of each participant was extracted from peripheral blood lymphocytes using the QIA amp DNA Blood Mini Kit (QIAGEN, Hilden, Germany). The proband underwent next-generation sequencing using a panel containing 101 monogenic hypertension-related genes (Supplementary Table 1). Candidate variants were predicted by in silico analysis using MutationTaster2 (http://www.mutationtaster.org) and were verified in the proband and his family members by PCR. Then these candidate variants were sought in 100 hypertensives and 100 healthy controls to evaluate whether the variations were common genetic polymorphisms. All PCR products were sequenced using an ABI Prism 377 DNA sequencer (Applied Biosystems, Foster City, CA, USA).

### In silico model of 11βHSD2

A computational model of 11βHSD2 was constructed to provide structural explanations for identified mutation sites in *HSD11B2*. Because the crystal structure for 11βHSD2 is not available, we chose a homologous crystal (PBD: 3HFG) [13], which was used as a template to construct a model of 11βHSD2. Modeling was performed using the Modeller program (Version 9.19) and optimized by the Amber 14.0 [14] software package with the ff14SB force field [15] based on molecular dynamics simulation. Stereochemical properties were evaluated using the program PROCHECK (Version 3.5.4, Cambridge UK) and Verify 3D [16]. The final model was chosen on the basis of low-energy conformation with no steric clashes between side chains.

### Systematic review

Literature from the PubMed database was searched from 1977 to February 2020 for reports about AME using English terms “apparent mineralocorticoid excess syndrome” or “apparent mineralocorticoid excess” (Supplementary Fig. 1). A secondary search checked for further manuscripts with clinical details. Cases without clinical or genetic profiles were excluded. We also searched the Human Gene Mutation Database (www.hgmd.org) to obtain *HSD11B2* mutation data. Summarized data are shown as medians or frequencies, and Spearman correlations were used to determine the association between genotypic status and clinical parameters by IBM SPSS 20 statistical software (IBM Inc, NY, USA). Values of *P* < 0.05 were considered statistically significant.

## Results

### Clinical features

The clinical characteristics of subjects in this family are presented in Table 1. During hospitalization, the proband suffered from elevated BP (170/110 mmHg), decreased serum potassium (2.50 mmol/L), and suppressed PAC (1.6 ng/dL). Other biochemical results revealed that he had hypernatremia, metabolic alkalosis, and proteinuria. Echocardiography showed enlargement of the left atrium (anteroposterior diameter, 39 mm) and ventricle (end-diastolic diameter, 56 mm), and mild mitral regurgitation. Computed tomography scanning found multiple calcinoses and small cysts in the liver and both kidneys, but no abnormalities in adrenal glands or renal arteries. Five relatives (subjects I-2, I-4, II-2, II-4, and II-5) had varying degrees of late-onset hypertension, but no hypokalemia, hyporeninemia, or hypoaldosteronism. Subjects II-1, II-6, and III-1 were normotensives.

**Table 1:** Clinical features of all participants in this family

Subjects	Gender	Age, years	Onset age of hypertension, years	Maximum BP, mmHg	K^+^, mmol/L	Na^+^, mmol/L	PRC, μIU/ml	PAC, ng/dL	Metabolic alkalosis	Nephrocalcinosis	Genotype
Grandmother I-2	F	69	50	160/90	3.59	138.35	7.2	18.8	N	N	c.343_348del/-
Grandmother I-4	F	64	55	145/85	3.77	137.24	15.8	13.7	N	N	c.1099_1101del/ -
Uncle II-1	M	47	–	132/84	4.97	144.77	38.3	27.4	N	N	–/–
Uncle II-2	M	45	31	170/103	4.02	145.47	6.4	8.2	N	N	c.343_348del/-
Father II-4	M	42	30	165/100	3.63	140.64	11.7	5.3	N	N	c.343_348del/-
Mother II-5	F	41	35	160/90	3.86	141.42	9.3	13.1	N	N	c.1099_1101del/-
Uncle II-6	M	38	–	128/82	4.15	142.50	33.2	27.9	N	N	–/–
Cousin III-1	M	20	–	125/80	4.37	146.90	35.0	25.6	N	N	–/–
Proband III-2	M	17	15	180/110	2.50	147.61	12.2	1.6	Y	Y	c.343_348del/c.1099_1101del

*F* female, *M* male, *BP* blood pressure, *K*^*+*^ serum potassium level (normal range, 3.5–5.3 mmol/L), *Na*^*+*^ serum sodium level (normal range, 137–147 mmol/L), *PRC* plasma renin concentration (normal range, 4.4–46.1 μIU/mL), *PAC* plasma aldosterone concentration (normal range, 3.0–35.3 ng/dL)

The proband received tailored management for AME. He maintained a healthy lifestyle, including a low-salt diet and moderate levels of exercises, and was treated with spironolactone (40 mg/day) and low-dose dexamethasone (1.5 mg/day) for 3 months. Follow-up results at 1 month showed a notable improvement in BP (140/90 mmHg) and serum potassium level (3.09 mmol/L), and normal BP (125/85 mmHg) and serum potassium (4.38 mmol/L) were achieved after 3 months. Three months later, dexamethasone was tapered off until disused and replaced by verapamil (120 mg/day) and spironolactone (40 mg/day), and the proband remained normotensive and normokalemic.

### Genetic analysis

Next-generation sequencing in the proband identified novel compound heterozygous variants: c.343_348del and c.1099_1101del (Fig. 1b, c). A paternally inherited frameshift variant, c.343_348del, was detected in exon 2 of *HSD11B2*, resulting in a deletion of Glu115 and Leu116 and production of a truncated 11βHSD2 protein. Another maternally inherited variant, c.1099_1101del, was identified in exon 5 of *HSD11B2*, resulting in a Phe367 deletion. Family screening identified that subjects I-2, II-2, and II-4 had the heterozygous variant c.343_348del, and that subjects I-4 and II-5 carried the heterozygous variant c.1099_1101del. Neither of the identified variants was detected in 100 hypertensives or 100 healthy controls, indicating that these were not common genetic polymorphisms.

Multiple sequence comparisons showed that the identified variants were conserved (Fig. 1d). They were not found in the Genome Aggregation Database (https://gnomad.broadinstitute.org) or the 1000 Genomes Project database (http://browser.1000genomes.org). In silico analysis using MutationTaster2 (http://www.mutationtaster.org) predicted that the compound variants are disease-causing.

### Protein modeling

**Fig. 2:**
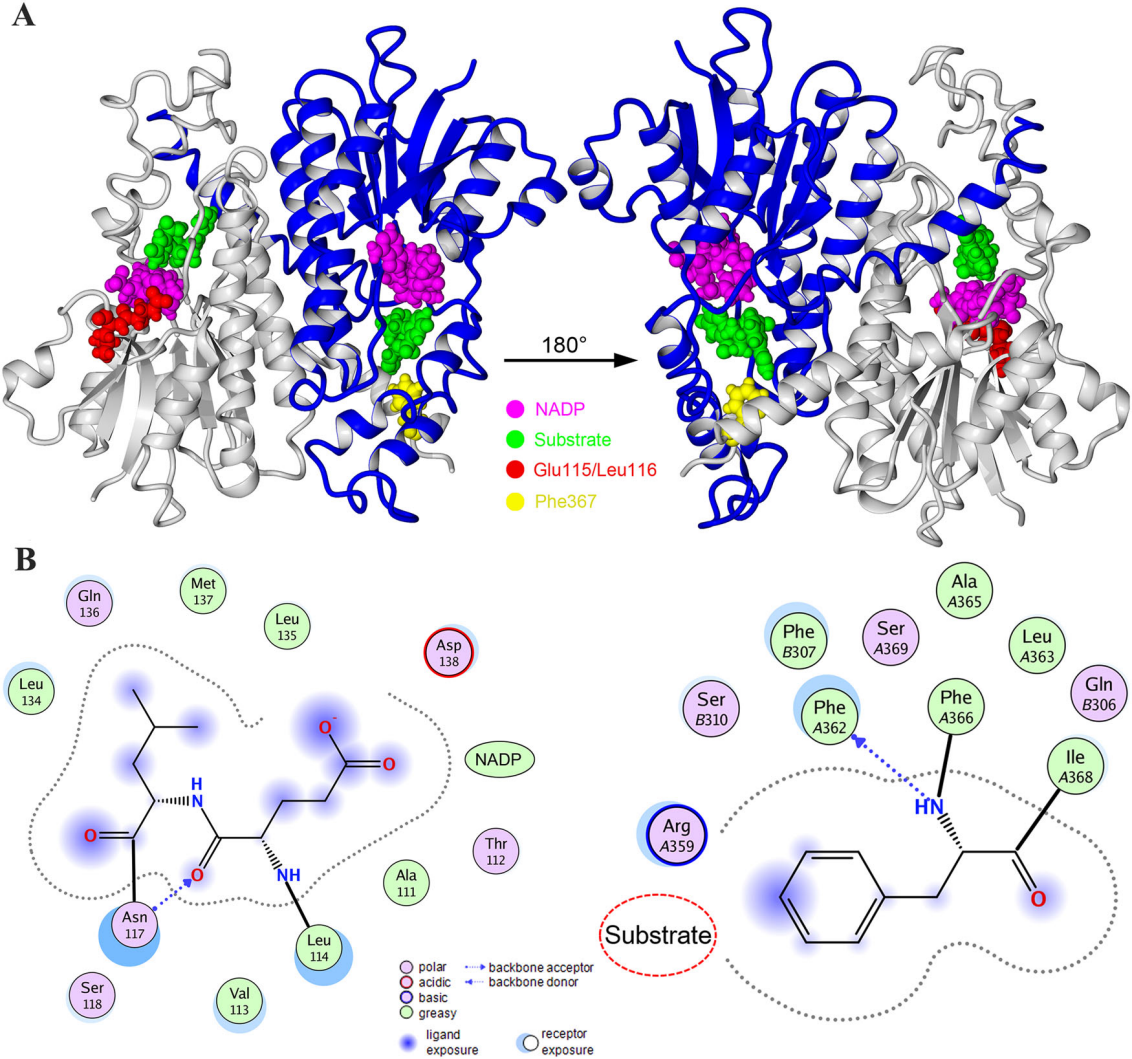
In silico three-dimensional modeling of 11βHSD2. **a** Amino acid residues Glu115/Leu116 and Phe367 are close to the cofactor (NADP) and substrate-binding sites. **b** Glu115/Leu116 and Phe367 are predicted to anchor NADP and substrate in the correct position via indirect hydrogen bonds and hydrophobic interactions

The protein model predicted the effects of truncated 11βHSD2 on structure and function. Amino acid residues p.Glu115/p.Leu116 are close to the nicotinamide adenine dinucleotide phosphate (NADP) binding site and p.Phe367 is not far from the substrate-binding site (Fig. 2a). Hydrogen bonds are formed between p.Glu115/p.Leu116 and p.Asn117, as well as p.Phe367 and p.Phe362 (Fig. 2b). In addition, the side chains of p.Leu116 and p.Phe367 generate a robust hydrophobic interaction together with surrounding amino acid residues. Hence, mutant 11βHSD2 may not only disturb the interaction between nearby amino acid residues but also affect the stability of NADP and the substrate in the binding site, thus leading to the loss of enzyme activity.

### Systematic review of genetic and clinical features of AME

**Fig. 3:**
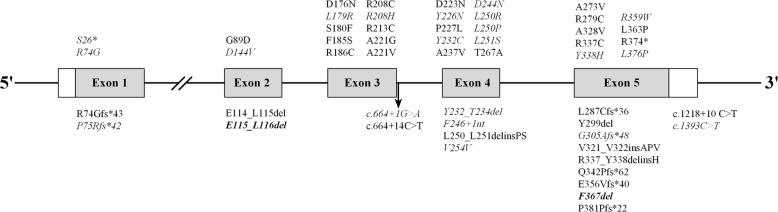
Location of 54 *HSD11B2* mutations described in the previous and present studies. Gray boxes indicate exons, and connecting lines represent introns. Italic mutations indicate the heterozygous state, and the identified compound mutations in this family are shown in bold

A total of 101 patients with AME were included in the systematic review: the 100 reported cases (Supplementary Fig. 1) and the proband in this study. Fifty-four *HSD11B2* mutations associated with AME were identified, including 31 homozygous mutations and 23 heterozygous mutations in various compound forms (Fig. 3). Most mutations were located within exons 3–5.

Clinical features of AME are summarized in Table 2. The median age at genetic diagnosis was 4 years (interquartile range, 1–11 years). Patients were usually born to consanguineous parents (80.6%), and 73% had a low birth weight (median, 2.1 kg). Hypertension was present in 99% of patients, hypokalemia in 93.7%, hypoaldosteronism in 95.3%, hyporeninemia in 87.3%, metabolic alkalosis in 76.3%, and nephrocalcinosis in 79.4%. Only 17.8% of cases carried compound mutations in *HSD11B2*. Compared with patients with compound genotypes, AME patients carrying homozygous mutations had a significantly low birth weight (*r* = 0.285, *P* = 0.02).

**Table 2:** Systematic review data of clinical and genetic characteristics of 101 patients with apparent mineralocorticoid excess syndrome caused by *HSD11B2* mutations

Parameters	Summary data^a^	Number of cases available
Male, *n* (%)	57	101
Consanguineous marriage of parent, *n* (%)	54	67
Low birth weight, *n* (%)	54	74
Median birth weight, kg	2.1 (1.9–2.3)	66
Median age at genetic diagnosis, years	3.8 (1.0–10.6)	98
Hypertension, *n* (%)	98	99
Median systolic/diastolic BP, mmHg	148/91 (135–170/80–110)	98
Hypokalemia, *n* (%)	89	95
Median serum or plasma K^+^, mmol/L	2.6 (2.2–2.9)	94
Hypoaldosteronism, *n* (%)	61	64
Hyporeninemia, *n* (%)	55	63
Metabolic alkalosis, *n* (%)	45	59
Median ratio of (THE + alloTHF)/THE	13.6 (6.2–28.0)	71
Nephrocalcinosis, *n* (%)	50	63
LVH, *n* (%)	21	30
Compound heterozygous genotype, *n* (%)	18	101

*BP* blood pressure, *K*^*+*^ potassium level, *LVH* left ventricular hypertrophy, *(THE* *+* *alloTHF)/THE* the ratio of urinary tetrahydrocortisol plus alloTHF to tetrahydrocortisone

^a^Categorical data were expressed as frequencies, and skewed distributed data were presented as medians (25th–75th percentile)

## Discussion

In this study, we identified novel compound heterozygous mutations (c.343_348del and c.1099_1101del) in *HSD11B2*, which were associated with AME in a Chinese family. The proband’s symptoms show that these mutations are causative of AME, and this is supported by structural modeling and in silico analysis. Moreover, we conducted the first known systematic review to analyze genotype–phenotype correlations in AME.

Patients with AME may be either homozygous or compound heterozygous carriers of *HSD11B2* mutations [17]. It is noteworthy that previously described mutations are mostly missense mutations clustering in exons 3, 4, and 5 [10]. Our systematic review recorded 54 homozygous or compound heterozygous mutations, including 31 (57%) missense mutations and 42 (78%) mutations located in exons 3–5. Other genetic aberrancies including nonsense, splicing, insertion, and deletion mutations were also recorded, but at a lower rate. The identified compound heterozygous mutations were situated in exons 2 and 5, and resulted in deletions of amino acid residues causing truncated 11βHSD2.

Previous cases with two identical heterozygous mutations from different families have been described. Ferrari et al. reported a girl with a homozygous mutation (c.343_348del) in *HSD11B2*, resulting in hypertension and suppressed plasma renin levels [7]. Morineau et al. described a French boy with compound heterozygous mutations (c.431 A > T and c.1099_1101del) in *HSD11B2* causing severe hypertension, low to normal serum potassium, low plasma renin, and aldosterone levels, as well as markedly reduced 11βHSD2 activity [10]. This study is the first report of the novel form of compound mutations (c.343_348del and c.1099_1101del) in *HSD11B2* in a Chinese teenager with AME.

Genetic mechanisms could account for AME caused by the identified compound mutations in the proband. Loss of catalytic activity and affinity for the substrate and cofactor is thought to be essential mechanisms resulting in a loss of enzymatic function and development of AME [18]. In the kidney, the non-selective MR has a similar affinity for aldosterone and cortisol, while circulating levels of cortisol are 100- to 1000-fold higher than healthy controls [19, 20]. 11βHSD2 not only catalyzes cortisol into inactive cortisone, but also generates the co-substrate nicotinamide adenine dinucleotide, thereby preventing cortisol-occupied MR from acting as aldosterone mimics [21]. The two heterozygous deletions identified in this study may impair the catalytic activity of 11βHSD2, decreasing the conversion of cortisol to cortisone. In addition, mutations of *HSD11B2* residues that form the substrate or coenzyme binding pocket could eliminate enzyme activity [22]. The homozygous mutation c.343_348del in *HSD11B2* has been identified in the cofactor binding domain with an apparent increased affinity for cortisol [23]. According to our structural modeling assessment, we postulate that the identified compound mutations disturb the substrate and coenzyme binding pocket. Therefore, intracellular cortisol would bind and overstimulate the MR, causing renal sodium reabsorption and potassium and bicarbonate excretion, resulting in the typical clinical features of hypertension and hypokalemic metabolic alkalosis. The development of nephrocalcinosis and renal cysts may be associated with long-standing hypokalemia [24].

Previous studies have revealed close genotype-structure-phenotype correlations in AME [11, 18, 25]. AME can be severe or mild, mainly depending on absent or residual 11βHSD2 activity caused by *HSD11B2* mutations [6]. Patients carrying *HSD11B2* homozygous mutations resulting in little or no 11βHSD2 activity usually present with severe phenotypes of AME in early childhood, whereas heterozygous patients with mutations resulting in partial 11βHSD2 activity may present with mild forms of AME in late adolescence or early adulthood [10, 22]. Our systematic review showed that homozygous *HSD11B2* mutations were significantly associated with low birth weight. In the case of compound heterozygosity, the clinical phenotypes result from the combined effects of two mutations [11]. However, the proband’s relatives carrying a single heterozygous mutation only presented mild to moderate hypertension in adulthood. This nonclassic AME may reflect haploinsufficiency of *HSD11B2* [26, 27]. Yau et al. found that mutations causing severe AME enhance dimerization, disrupt substrate or coenzyme binding sites, or severely impair structural stability of the enzyme [11]. Our protein modeling analysis predicted that the identified compound mutations probably impacted the substrate and coenzyme binding sites concurrently. Furthermore, the variability in clinical manifestations might be associated with epigenetics, sodium, and environmental factors [26, 28].

Because the age of AME presentation varies and complications can be severe [29], early diagnosis and treatment are vital to prevent end-organ damages [30]. As a congenital disorder, AME usually presents in early childhood [29]. Our systematic review revealed a median age at genetic diagnosis of 3.8 years (interquartile range, 1.0–10.6 years), with 25.5% of patients diagnosed within the first year of life. However, our proband presented and diagnosed with a moderate to severe form of AME in late adolescence. Severe hypertension and hypokalemic alkalosis are associated with end-organ damage in AME, especially affecting cardiovascular and nervous systems, kidneys, and retina [3]. Variability in biochemical parameters (the ratio of cortisol to cortisone) is related to the underlying genetic defect [31], so genetic testing is a precise and effective tool to detect *HSD11B2* mutations [6, 32]. Hence, our study also highlights the utility of next-generation sequencing for diagnosing AME even where enzymatic characterization is unavailable.

The goal of AME treatment is to control BP and correct electrolyte disturbance [33–35]. Therapeutically, it is important to reduce dietary sodium [36]. The proband was treated with spironolactone, low-dose dexamethasone, and verapamil for various periods. Spironolactone, as a mineralocorticoid antagonist, is required to block the MR from cortisol activation [33]. Dexamethasone relieves excessive MR activation from cortisol by the suppression of peripheral cortisol secretion [37]. However, dexamethasone cannot correct hypertension and hypokalemia in AME patients, and long-term medication has major adverse effects [37]. Therefore, the treatment of AME with low-dose dexamethasone (1.5–2 mg/day) should be initiated in a monitored setting [35]. Use of a calcium channel blocker as an adjunctive treatment to control hypertension was reported to be helpful [24]. Other medicines have also been used to treat AME, such as potassium supplementation [6] and/or epithelial sodium channel inhibitors [37]. Nevertheless, comprehensive and effective management of AME patients still needs further investigation with larger sample sizes and long-term follow-up times.

In conclusion, we identified novel compound heterozygous mutations in *HSD11B2* in a non-consanguineous family with AME. To the best of our knowledge, this is the second reported AME case in the Chinese population. Genetic testing can provide a definitive diagnosis for AME patients, which can prevent secondary organ damage together with specific patient management. Further in vitro expression studies should evaluate the enzymatic activity of mutant 11βHSD2 proteins to verify the severity of the compound mutations.

## Supplementary information

Supplemental Table 1

Supplemental Figure
