# Supplementary material for: Apparent mineralocorticoid excess caused by novel compound heterozygous mutations in HSD11B2 and characterized by early-onset hypertension and hypokalemia
Source: Endocrine. 2020 Aug 20;70(3):607–15. doi: 10.1007/s12020-020-02460-9 (PMC7674368; doi:10.1007/s12020-020-02460-9)
Supplement: Supplementary file 1 — Supplemental Table 1 [file 12020_2020_2460_MOESM1_ESM.doc]

**Supplemental Table 1**. List of 101 genes was selected to perform the panel design for next-generation sequencing in this study.

| *ACE* | *BBS7* | *CYP21A2* | *IKBKAP* | *NR3C1* | *SDHC* |
| --- | --- | --- | --- | --- | --- |
| *ADD1* | *BBS9* | *CYP3A5* | *KCNJ1* | *NR3C2* | *SDHD* |
| *AGT* | *BMPR2* | *ECE1* | *KCNJ5* | *PDE11A* | *SELE* |
| *AGTR1* | *CACNA1D* | *EGLN1* | *KCNMB1* | *PDE3A* | *SERPINA6* |
| *AIP* | *CACNA1H* | *EGLN2* | *KIF1B* | *PDE8B* | *SLC12A1* |
| *ALMS1* | *CCND1* | *EPAS1* | *KLHL3* | *PLIN1* | *SLC12A3* |
| *APOA1* | *CDKN1B* | *FGA* | *LYZ* | *PRKAR1A* | *STOX1* |
| *ARL6* | *CEP19* | *FH* | *LZTFL1* | *PTGIS* | *SUMO4* |
| *ARMC5* | *CEP290* | *FMO3* | *LZTR1* | *RET* | *TMEM127* |
| *ATP1B1* | *CLCNKB* | *FN1* | *MAX* | *RGS5* | *TNXB* |
| *BBIP1* | *COL4A1* | *GDNF* | *MEN1* | *SCNN1A* | *TRIM32* |
| *BBS1* | *COL4A2* | *GNAS* | *MKKS* | *SCNN1B* | *TTC8* |
| *BBS10* | *COL4A3* | *GNB3* | *MKS1* | *SCNN1G* | *VHL* |
| *BBS12* | *CORIN* | *GUCY1A3* | *MYH8* | *SDCCAG8* | *WDPCP* |
| *BBS2* | *CUL3* | *HMBS* | *NF1* | *SDHA* | *WNK1* |
| *BBS4* | *CYP11B1* | *HSD11B2* | *NOS2* | *SDHAF2* | *WNK4* |
| *BBS5* | *CYP17A1* | *IFT27* | *NOS3* | *SDHB* |  |
