# Supplementary figures and images for: Apparent mineralocorticoid excess caused by novel compound heterozygous mutations in HSD11B2 and characterized by early-onset hypertension and hypokalemia
Source: Endocrine. 2020 Aug 20;70(3):607–15. doi: 10.1007/s12020-020-02460-9 (PMC7674368; doi:10.1007/s12020-020-02460-9)

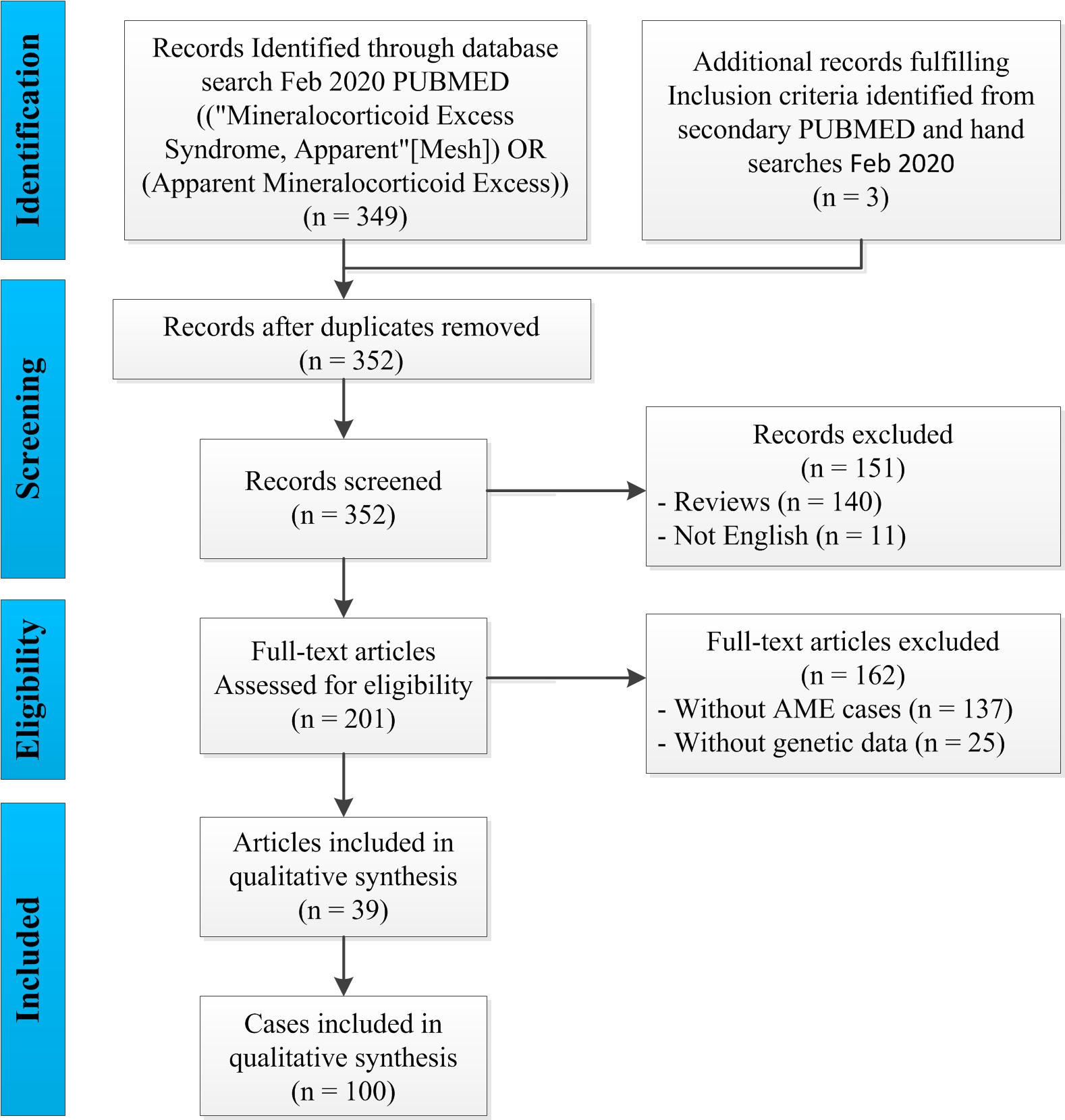

Supplement: Supplementary file 2 — Supplemental Figure [file 12020_2020_2460_MOESM2_ESM.tif]
